# Reliability Evaluation of the Data Acquisition Potential of a Low-Cost Climatic Network for Applications in Agriculture

**DOI:** 10.3390/s20226597

**Published:** 2020-11-18

**Authors:** Sergio Trilles, Pablo Juan, Carlos Díaz-Avalos, Sara Ribeiro, Marco Painho

**Affiliations:** 1 Institute of New Imaging Technologies, Universitat Jaume I, Av. Vicente Sos Baynat s/n, 12071 Castellón de la Plana, Spain; 2 IMAC, Mathematics Department, Universitat Jaume I, Av. Vicente Sos Baynat s/n, 12071 Castelló de la Plana, Spain; juan@uji.es; 3 Department of Probability and Statistics, Institute of Research in Applied Mathematics and Systems, Universidad Nacional Autónoma, 04510 Ciudad de México, Mexico; zhangkalo@gmail.com; 4 Nova Information Management School (NOVA IMS), Universidade Nova de Lisboa, 1070-312 Lisbon, Portugal; sribeiro@novaims.unl.pt (S.R.); painho@novaims.unl.pt (M.P.)

**Keywords:** low-cost sensors, homogenisation, cross-correlation, climatic conditions

## Abstract

Temperature, humidity and precipitation have a strong influence on the generation of diseases in different crops, especially in vine. In recent years, advances in different disciplines have enabled the deployment of sensor nodes on agricultural plots. These sensors are characterised by a low cost and so the reliability of the data obtained from them can be compromised, as they are built from low-confidence components. In this research, two studies were carried out to determine the reliability of the data obtained by different *SEnviro* nodes installed in vineyards. Two networks of meteorological stations were used to carry out these studies, one official and the other professional. The first study was based on calculating the homogenisation of the data, which was performed using the Climatol tool. The second study proposed a similarity analysis using cross-correlation. The results showed that the low-cost node can be used to monitor climatic conditions in an agricultural area in the central zone of the province of Castelló (Spain) and to obtain reliable observations for use in previously published fungal disease models.

## 1. Introduction

Knowledge of current meteorological conditions is an essential piece of information to make weather forecasts [1], detect possible disease outbreaks on agricultural land [2] or to predict spatial trends in air pollution [3], as well as traffic [4] levels in cities, among others. Since climate change has become recognised as a severe problem in recent years, weather monitoring has gained importance [5]. This has led to the deployment of a significant number of weather monitoring stations operated by government agencies in different countries.

An example of such official weather stations is the Automated Weather Data Acquisition System (*AMeDAS*) developed by the Japan Meteorological Agency [6]. To date, *AMeDAS* has installed stations at 1300 locations in Japan. These stations have sensors that monitor temperature, humidity, solar radiation, wind speed/direction, precipitation, atmospheric pressure and other phenomena. In Spain, the “Agencia Estatal de Meteorología” (*AEMET*) is responsible for maintaining almost 800 weather stations across the country [7]. The *AEMET*’s monitoring stations observe meteorological phenomena as well as solar radiation, ozone and several pollutants. In both monitoring systems, data accuracy is certified by the organisation responsible for the deployment and maintenance of the network. The data obtained are used for several purposes, including weather forecasting, disaster forecasting or crop modelling.

Official monitoring stations are expensive, mainly because they require complex installations. These features make them impossible to install in places that are difficult to access, or where installation and maintenance are simply not feasible. Wireless Sensor Networks (WSN) have been proposed [8,9] to address these cost and flexibility issues. One of the main objectives of WSN is cost reduction, and low-cost sensors are often used to build weather stations [10]. This proliferation of sensors together with the explosion of the paradigm of the Internet of Things has increased the ubiquity of sensorisation, which has caused a revolution that promises to create a newly interconnected “smart” world [11].

Agriculture is one of the most important fields in the use of these sensor networks because climatic conditions are one of the main threats promoting diseases in crops [12]. The orography and the environmental factors of the location where a vineyard smallholding is cultivated affect the state of the soil and the plants [13]. The temperature has an important influence during the growth and maturation vineyard states. Some vineyard diseases could appear during a specific range of temperatures. Among these diseases, we can list powdery mildew [14], downy mildew [15], black rot, or botrytis [2]. Air humidity is another phenomenon that has a high implication in (powdery and downy) mildew diseases. Another phenomenon with a high impact on diseases is precipitation. It can be used together with air humidity and temperature to follow different algorithms to detect fungal diseases such as downy mildew.

The incorporation of Information and Communication Technologies (ICT) through the smart farming [16] movement, along with the reduced cost of electronic components, has favoured the increase in meteorological monitoring stations in order to know first-hand the state of the smallholdings. This knowledge is especially decisive in the cultivation of vineyards [17]. Meteorological monitoring within the vineyard parcels is vital to detect and anticipate possible fungal diseases. In particular, the phenomena of temperature, humidity and precipitation are the most critical factors to control possible fungal diseases. Examples of these vineyard diseases are downy mildew, powdery mildew, black rot and botrytis [2].

During the last decade, different initiatives from ICT and more concretely from the Internet of Things (IoT) have been applied in vineyard smallholdings to detect the most predominant diseases in this cultivation. All these proposals follow the same objective, monitor the main environmental factors listed above. One example of these initiatives is Vinesens, where the author developed and deployed different nodes with the primary environmental sensor (temperature, air and soil humidity, and precipitation). The data are collected by a central server to visualise and control these parameters using alerts [13]. A node with six environmental sensors (air humidity and temperature, soil moisture and temperature, the water level in soil sensor, PH value sensor and dew sensor) was created in [18] to control the good conditions parameters value for the growth of ten diseases on grapes. More recent work is presented in [19], wherein the nodes were designed with temperature and humidity sensors, and the main functionality from the server-side is to support different prediction strategies of grapevine contamination by Downy Mildew. Finally, out of academia, the company SmartVineyards provides a precision viticulture system capable of monitoring in-situ climatic conditions and offering disease predictions and alerts to the users [20]. The same initiative can be used to plan the grape production activities and calculate the negative impact on vine production.

In [21] the authors proposed a design for a low-cost monitoring platform for monitoring meteorological phenomena called *SEnviro* [22]. Similar to those discussed above, this platform is used for monitoring within the vineyard smallholdings, and the captured observations are employed to detect possible cases of the above-mentioned diseases. Due to the price of the meteorological sensors that are incorporated (which will be described later on), they are considered low-cost components. The reliability of the data generated by these low-cost sensors has already been shown to be compromised in numerous studies [23]. However, the data produced by these sensors are often contaminated by events other than variability, such as errors in taking or transmitting them, as well as changes in the instrument used, in the location of the node or in its environment. These alterations in the series of observations, called inhomogeneities, mask the real changes in the climate and cause the study of the series to lead to erroneous conclusions [24]. For years homogenisation methodologies have made it possible to eliminate or reduce these unwanted alterations as much as possible. Initially, they consisted in comparing a problem series with a supposedly homogeneous one. However, this is a rather hazardous assumption and, thus, more recent research studies build a reference series from the average of others selected for their proximity or high correlation, thus diluting their possible inhomogeneities. As this does not guarantee that the reference series is homogeneous, other methods compare all available series in pairs, so that repeated detection of an inhomogeneity allows identification of the wrong series [25,26].

With this background, the main objective of this study was to evaluate the reliability of the observations produced by *SEnviro* nodes. More particularly, this evaluation was carried out on the meteorological phenomena used in the detection of vine diseases (temperature, humidity and rainfall). To achieve this goal, two other meteorological sensor networks have been used. The first is an official state-level meteorological monitoring network, called *AEMET* and the second is called *AVAMET*, which houses professional meteorological stations and has a regional scope of application. This evaluation was performed using the meteorological data captured by the three networks during the 2018 wine campaign. Two complementary approaches were defined. The first, the Climatol tool [27], was used to homogenise the meteorological data, and the quotient between the range of phenomena and its square root was applied in order to achieve a better homogenisation of the data and to ensure they are more comparable. The second was a similarity analysis performed between the closest stations using cross-correlation [28].

The rest of the paper is organised as follows. Section 2 introduces all the sensor networks and models used to carry out this evaluation. Section 3 shows the results obtained after applying the models and, in parallel, it discusses them with a small analysis of each one. The paper finishes with Section 4, which presents the conclusions and offers recommendations for future work.

## 2. Materials and Methods

This section describes all the materials and methods needed to perform the reliability analysis of the *SEnviro* low-cost nodes. The first subsection introduces each of the sensor networks used to perform the evaluation and the last part of this subsection details how the data integration process was carried out to prepare the data for analysis. The second subsection describes the methods used to perform the appraisal.

### 2.1. Meteorological Sensor Networks

#### 2.1.1. Low-Cost Sensor Network: *SEnviro* Network

**Figure 1:**
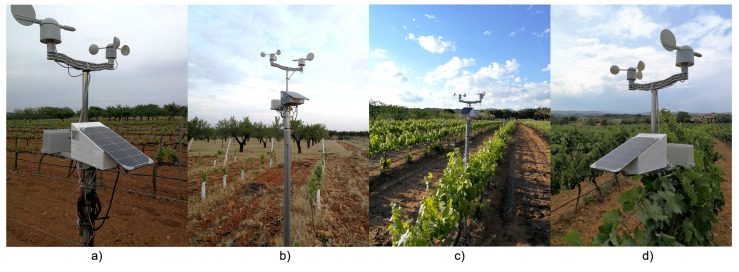
Some pictures of *SEnviro* node deployments in vineyards.

**Figure 2:**
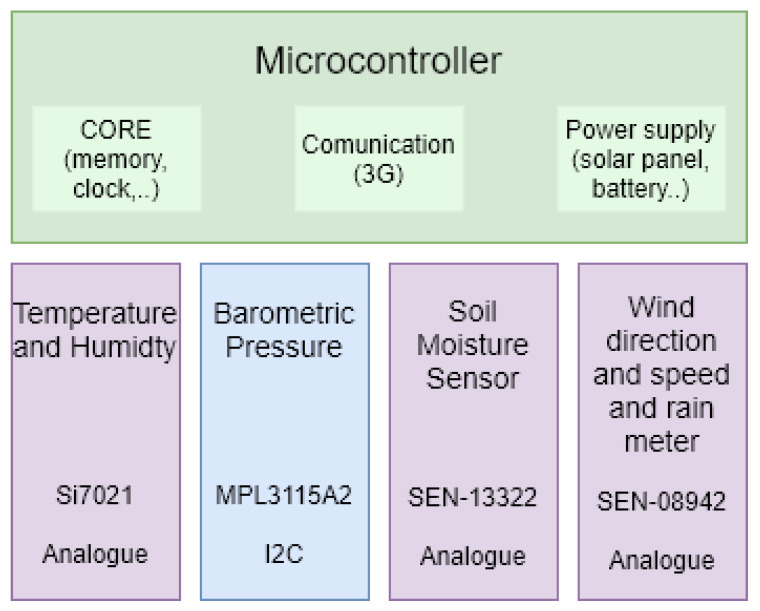
A representation of all environmental sensors included in a *SEnviro* node.

The main objective of the current work is to evaluate the reliability of a low-cost meteorological node called *SEnviro* [21]. This node is made using open hardware components (see Figure 1). Following the structure of categories defined in [10], the *SEnviro* node can be split into four categories (core, communication, sensors and power supply). All the components used to build a *SEnviro* node are properly described in a previous work [21]. The *SEnviro* node includes a 3G connection and a solar panel in order to make it an autonomous solution. These nodes contain sensors to monitor meteorological phenomena such as temperature, air/soil humidity, wind speed, wind direction, and rainfall. The phenomena are described in the following subsections, including the units, range and accuracy in each case (Figure 2).

- Temperature. Units: Centigrade; Range: (−10, 85); Accuracy: ±0.4 degrees (C)
- Humidity. Units: Percentage; Range: (0%, 80%); Accuracy: ±3 RH
- Barometric Pressure. Units: Hectopascal; Range: (500, 1100); Accuracy: ±0.04 hPa
- Soil Moisture. Units: Percentage; Range: (0%, 85%); Accuracy: ±0.5 RH
- Wind speed. Units: km/h; Range: N/A; Accuracy: N/A
- Wind direction. Units: Direction (degrees); Range: (0) North, (1) NE, (2) East, (3) SE, (4) South, (5) SW, (6) West, (7) NW and (−1) error; Accuracy: N/A
- Rain meter. Units: millilitres (mm); Range: N/A; Accuracy: N/A

**Figure 3:**
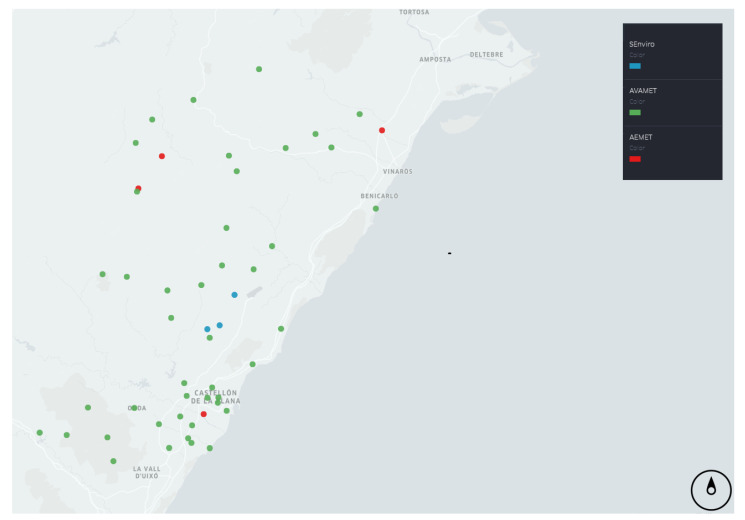
Map showing the location of each node from all sensor networks.

The data used to conduct this evaluation arrived from three different low-cost nodes stationed in vineyard smallholdings placed in the province of Castelló, in the Valencian Community, Spain (Figure 3). All *SEnviro* nodes are included in a circular bounding box with a diameter of 4 km. The study area is between 240 to 320 m above sea level. The predominant climate is local steppe. There is little rainfall throughout the year. This climate is considered BSk according to the Köppen–Geiger climate classification. It should be noted that the province of Castelló is an area of great climatic variety due to its mountainous orography [29,30], with high temperatures in summer and very low ones in winter, and an important difference between the coast and the interior. The average temperature in this zone is 14.00 degrees (C), and the approximate precipitation is 434 mm.

Table 1 adds more information about the different locations for each *SEnviro* node and specific characteristics about the smallholding (cultivation area or vine varieties). The smallholdings where the *SEnviro* nodes are located have in total of 55,000 square metres of area. The vine varieties in these areas are Monastrell with the first sensor, Merlot, Cabernet, Chardonnay and Syrah where the second one is, and Bonicaire vine variety for the third *SEnviro* sensor.

In the best case, these IoT nodes worked continuously and uninterruptedly for 140 days. Each node sent an observation every ten minutes during the 2018 wine season, from 1 April 2018 to 31 October 2018. In all, 20,160 observations were collected per node [31].

Each *SEnviro* unit has a price of € 256.45. The energy consumption of the node is 21,4625 mA in the normal energy mode. Using a battery with 2000 mA, the node can work continuously for 93.19 h without solar charging. Also, it supports a safe mode (without sending observations) to prolong battery life for more than 54 days.

#### 2.1.2. Official and Professional Networks: *AEMET* and *AVAMET*

As stated above, two other weather station networks located in the same study area have also been considered (Figure 3). These two networks are of different types. The first is *AEMET*, which is an official meteorology network supported by the Spanish government and deployed throughout the whole of Spain. The second network, called “Associació Valenciana de Meteorologia” (*AVAMET*), is a professional network, and its stations are distributed throughout the Valencian Community. Both weather networks are described in depth below.

##### AEMET

The “Agencia Estatal de Meteorología” (*AEMET*) is a Spanish state agency of which the fundamental objective is to provide meteorological services. This agency was created by *Real Decreto 186/2008*, on 8 February 2008, replacing the former National Institute of Meteorology. It is attached to the Ministry for Ecological Transition through the Secretary of State for the Environment. The network consists of more than 800 stations distributed throughout the country. AEMET stations are automatic (called EMAs in Spanish). An EMA station is generally composed of several sensors, a data acquisition and processing unit, a power supply system, and a communications unit. Sensors take data from the following phenomena: air temperature and humidity, precipitation and wind speed and direction. In addition, depending on the type of station, other phenomena are available: atmospheric pressure, visibility, solar radiation or soil temperature. The power supply system includes a solar panel and batteries. Some may be connected to the electrical network. To carry out communications, the station may have a modem/router. *AEMET* makes the information from its weather stations available to the general public via the open data portal (opendata.aemet.es).

##### AVAMET

The Valencian Association of Meteorology Josep Peinado (*AVAMET*) is a non-profit meteorological association that has as its main goal the monitoring of the weather conditions from all over the Valencian Community. The network has 444 stations throughout the Valencian region. *AVAMET* provides real-time weather data for the whole of the Valencian Community. The most available models are the Davis Vantage Vue and Davis Vantage Pro2. In addition to these predominant models, there are other kinds of stations models: Oregon, Sainlogic, Froggit and Netatmo. More concretely, forty-five professional stations have been used to perform this study. Only Davis Vantage Vue and Davis Vantage Pro2 stations have been selected to perform this study. These stations can measure temperature, air humidity, atmospheric pressure, wind direction and speed, and precipitation. These stations are energetically autonomous using a cell battery (or solar panel with a rechargeable battery), but they have a wireless console that needs to be connected in terms of energy. This console must also be connected directly to the energy current, and using a data logger connected to a computer to establish a connection to transfer the observations to the server.

#### 2.1.3. Data Preparation

The study area used in this work was Castelló, due to the fact that the *SEnviro* nodes are located on smallholdings in this province and, hence, only those weather stations have been considered. In the case of the *AEMET* network, there are five stations located in the area. For the *AVAMET* network, 63 stations have been installed in the province of Castelló, mainly within localities and on municipal property. Figure 3 shows the location of each of the stations that have been used for this study. The green stations are those belonging to the *AVAMET* network, the red ones belong to the *AEMET* network and the blue ones are part of the *SEnviro* network.

Of all the meteorological phenomena measurements provided by the weather stations, three specific aspects were selected, namely, temperature, humidity and precipitation. These three were selected because they are the essential meteorological phenomena in most models of detection of fungal diseases, especially in those listed above related to vine cultivation [2].

To compare the sensor data gathered, all data from all three sensor networks were analysed and homogenised. A daily frequency was chosen because the data provided by the *AEMET* network is published at that time rate. Data cleaning techniques were also applied in order to eliminate outliers and transmission errors in the *SEnviro* sensor network. All data were transformed into the format required by the models, and new files were generated to run the models described below. Not all the stations selected measure all three phenomena. If a station does not measure a phenomenon, it is no longer included in the analysis of this phenomenon. For example, the *AEMET* network does not offer the humidity value in any of the stations in the province. In the case of *AVAMET*, the number of stations that measure humidity is smaller than that of those that monitor the temperature. Finally, precipitation is available in three of the four *AEMET* stations and all *AVAMET* stations. The three phenomena analysed are available in the three units of the *SEnviro* node.

### 2.2. Methods

In this research work, we have proposed two different methods to validate the low-cost sensor network. The first method, using a library called Climatol [27], is carried out to test the homogeneity among the three sensor networks. The homogeneity of the climatological series is defined as the variation of a particular phenomenon from the study station concerning a reference station in the same area [32]. For this, an artificial series, combining several real series, is used to detect possible inhomogeneities. A comparison using these reference series can be made to validate the studying series, applying quotients or differences between both series. The quotients carry problems when the dividing series has null or close to zero value [33]. To solve this issue, the differences between the series to be compared are used, as previously published [34]. The main anomalies are divided into: (1) isolated errors, due to reading or transcription errors, or to specific errors in the sensor (automatic stations); (2) jumps in the series, attributable to changes in instrumentation or the installation conditions of the stations, and (3) gradual drifts in the series, due to progressive changes in the response of the sensors (recalibration) or in the environment (growth urban, changes in land use, etc.).

Climatol contains functions for quality control, homogenisation and the padding of missing data in a set of series of any climate phenomena with different rates (daily or monthly). Moreover, Climatol also served to carry out an exploratory study of the recorded data and allowed the data from the different weather stations to be compared and studied. This study was performed using sensor data provided by all the stations from all the sensor networks (Figure 3). From these data, in order to go further and compare the results between stations, the maximum and minimum of both temperature and humidity were obtained. Precipitation was calculated using the daily accumulated amounts.

As a second step, a direct comparative analysis between nearby stations was designed. Considering the data obtained from the different stations as a time series [35], this analysis was carried out using Cross-Correlation Function (CCF). For two time series *X* and *Y*, the sample cross correlation between them at lag *k* is defined as

(1) $$c_{k}\left(X,Y\right)=\frac{1}{n}\frac{\sum_{t=1}^{n-k}\left(Y_{t}-\bar{Y}\right)\left(X_{t-k}-\bar{X}\right)}{S_{X}S_{Y}}$$

where $S_{X}$ and $S_{Y}$ are the sample standard deviations for series *Y* and *Y* respectively. The cross-correlation function is a measure of linear association but it presents some drawbacks. Since it is a moment-related statistic, it is sensitive to outlying values. However, it has the advantage of being easy to interpret and to compute and can also be considered a similarity index [36]. High to moderate similarity is to be expected for some meteorological phenomena at close locations due to the existence of spatial correlation. For the similarity analysis, only the five *AVAMET* monitoring sites closest to our sensors were considered because we expected to observe a similarity between the different monitoring locations only at short to moderate distances.

**Figure 4:**
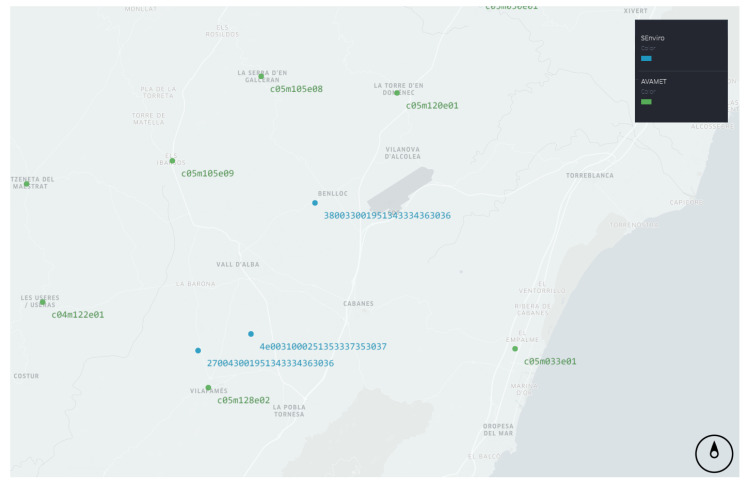
Map showing each of the *SEnviro* nodes and the locations of the closest *AVAMET* stations.

We used the values of the CCF to compare the similarity of the three *SEnviro* nodes and the closest *AVAMET* stations at different time lags (Figure 4) [28]. Although the time window for all the time series was the same, there were missing values in some series of the sensor stations due to technical problems that impeded the data from being recorded. Missing values in the original time series were present at different times for the different series. However, with the records that coincided in time it was possible to compute the cross-correlation functions between the different series [28]. In the comparisons we used only short lags because for larger values of *k* there are fewer $\left(x_{t},y_{t-k}\right)$ pairs and therefore higher uncertainty in the cross correlation estimates [35].

## 3. Results and Discussion

This section shows and discusses the results obtained after running both the models presented above.

### 3.1. Exploratory Analysis

**Figure A1:**
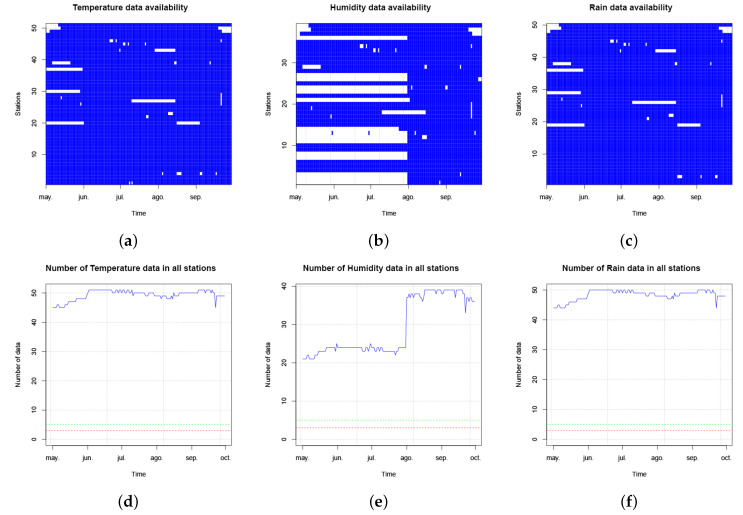
Exploratory analysis of data availability at all sensor stations and for all phenomena. (**a**) Temperature data availability at all stations. (**b**) Humidity data availability at all stations. (**c**) Precipitation data availability at all stations. (**d**) Number of temperature data at all stations. (**e**) Number of humidity data at all stations. (**f**) Number of precipitation data at all stations.

The first step in our study was to check data availability for the analysis and comparison of the network data. This included checking the availability of measurements of the different variables at all the stations and days. As we have indicated in Section 2.1.3, the data for each of the variables considered in the analysis have missing values scattered along time in all the stations for the three networks used in this study, and data for some variables may only be available for a reduced number of dates. Figure A1 shows the availability and number of values for temperature, humidity and precipitation. Figure A1a–c show the presence and distribution of missing values in the databases across time. For the data to be valid, there should be five or more measurements for a given variable available at each time step, or a minimum of three levels marked with dashed green and red lines on the right side of the Figure A1d–f. These requirements were met by the databases from the three station networks.

All the stations included have some values for all the selected phenomena, because otherwise the station is eliminated from that particular analysis. If we analyse the availability of the temperature data (Figure A1a,d), like the precipitation data (Figure A1c,f), they are complete across the entire working range. Regarding the humidity phenomenon (Figure A1b,e), there were no measured values in the first sections of the period observed, mostly at *AVAMET* network stations.

#### 3.1.1. General Range and Distribution

**Figure A2:**
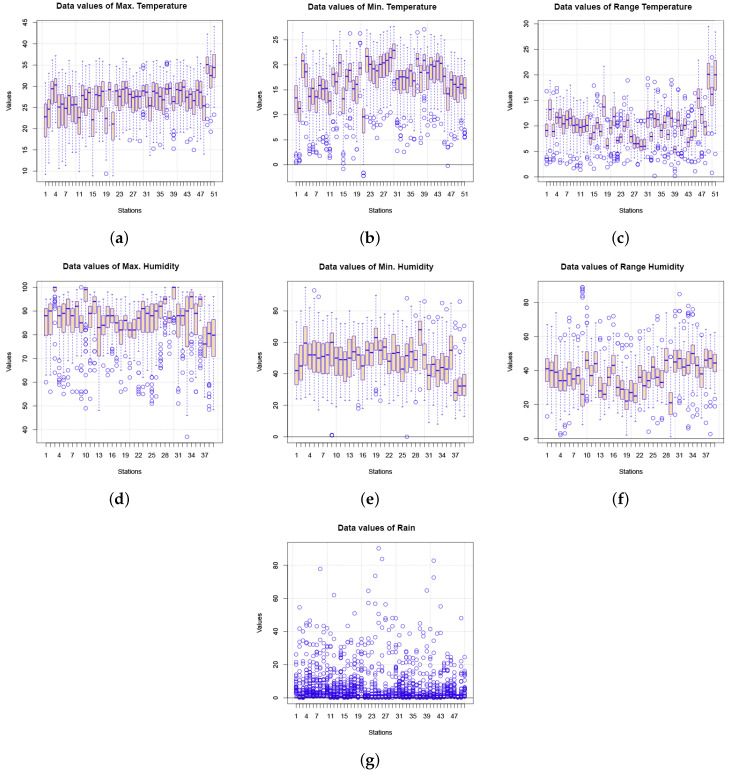
Temperature (minimum, maximum and range), humidity (minimum, maximum and range) and precipitation data values boxplots. (**a**) Data values of maximum temperature at all stations. (**b**) Data values of minimum temperature at all stations. (**c**) Data values of temperature range at all stations. (**d**) Data values of maximum humidity at all stations. (**e**) Data values of minimum humidity at all stations (**f**) Data values of humidity range at all stations. (**g**) Boxplots for precipitation data values.

**Figure A3:**
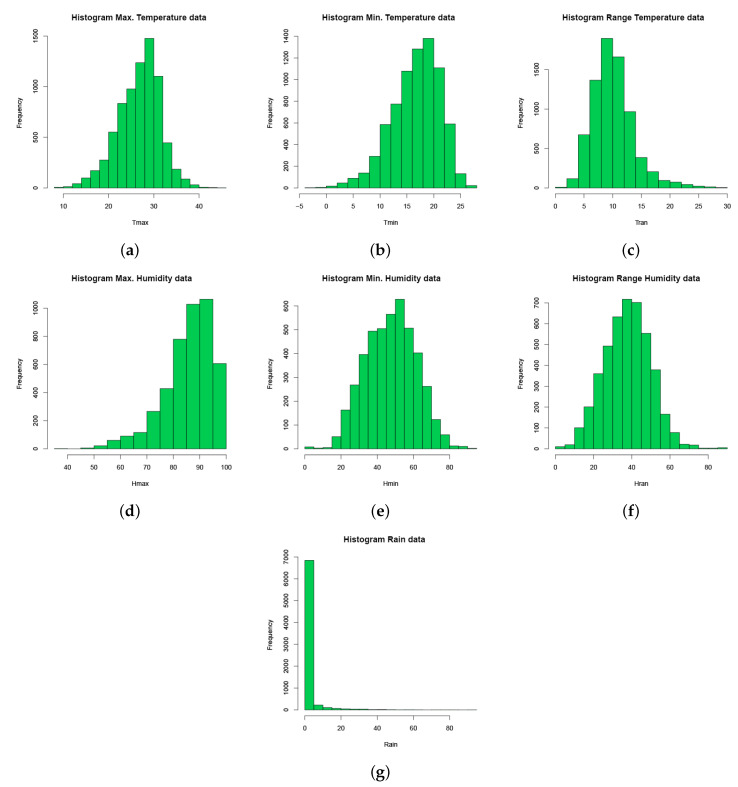
Temperature (minimum, maximum and range), humidity (minimum, maximum and range) and precipitation values histograms. (**a**) Maximum temperature values histogram. (**b**) Minimum temperature values histogram. (**c**) Range temperature values histogram. (**d**) Maximum humidity values histogram. (**e**) Minimum humidity values histogram. (**f**) Range humidity values histogram. (**g**) Precipitation values histogram.

This section discusses the box plots of the data at each station and the histograms of all the data (Figure A2 and Figure A3). The plots show the presence of anomalous values at stations 49–51, in particular for maximum temperature (Figure A2a) and temperature range (Figure A2c) and minimum humidity (Figure A2e). Regarding the temperature (Figure A2a–c), it can be seen in the measures that in the case of the minimum temperature (Figure A2b) there is not much difference between the different stations. In contrast, in maximum temperatures (Figure A2a) there is a clear difference between the *SEnviro* stations and the others. However, the ranges of variations are similar, from minimum to maximum. Unlike humidity, for temperature, the ranges are larger in the last three stations (Figure A2c). Both the maximum and minimum humidity of stations c06m021e02, c06m032e01 and c06m032e0 (*AVAMET*) are lower than the others (Figure A2d,e). Conversely, as we can see in Figure A2f, the last three are not different from the others. There are only small variations in the values from the different stations concerning the amount of rain (Figure A2g).

Figure A3 shows the overall data distribution of each measure, and thus the variation in the values of each of them. The frequency histograms for each climatic factor show that the frequency distribution for the values of the different variables (climatic factors) is unimodal and symmetric, except for some variables. Most of the data are distributed in the form of a Gaussian distribution, with some values lying towards the extremes (values scattered to the right in temperature range, Figure A3c) except in cases where we have many small values. Maximum humidity (Figure A3d) tends to have large values, such as an exponential distribution. In contrast, rain distribution (Figure A3g) tends towards a Gamma distribution with many small values for the number of days without rain. These graphs can help us when it comes to modelling the variables (phenomena) and to know what a priori distribution they present. The lack of multimodality in the histograms suggests that the values for the different variables in the study area are relatively homogeneous, although formal homogeneity tests are advisable.

#### 3.1.2. Correlograms, Dendrograms and Cluster Groupings

**Figure A4:**
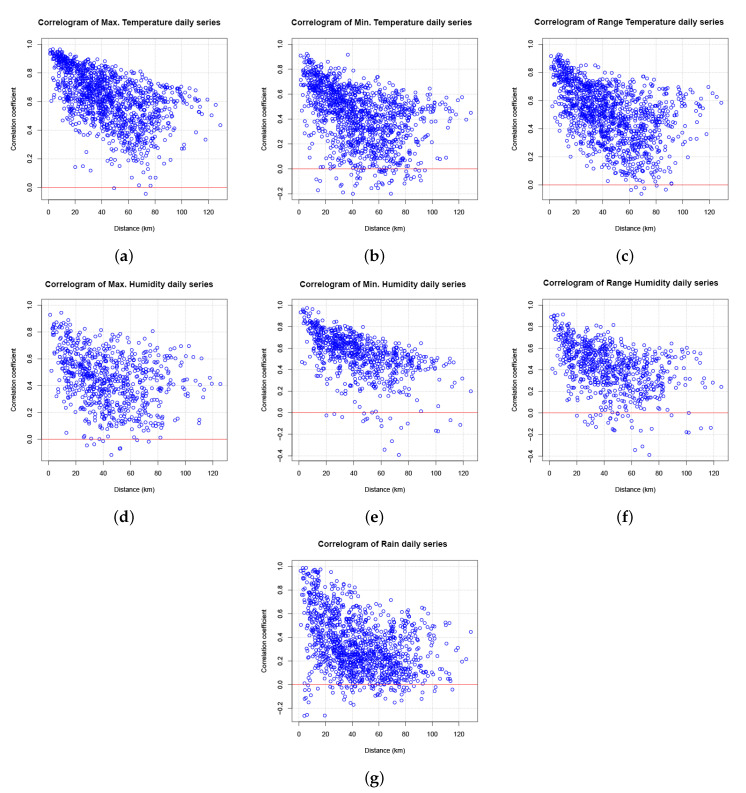
Temperature (minimum, maximum and range), humidity (minimum, maximum and range) and precipitation correlograms. (**a**) Maximum temperature values correlogram. (**b**) Minimum temperature values correlogram. (**c**) Range temperature values correlogram. (**d**) Maximum humidity values correlogram. (**e**) Minimum humidity values correlogram. (**f**) Range humidity values correlogram. (**g**) Precipitation values correlogram.

The graphs in Figure A4 show the correlations with the arrangement of the stations, an essential element to be able to correlate the values of the stations for each of the measures. We should highlight the more significant variability by increasing the distance between stations in the case of maximum humidity (Figure A4d) and especially in the case of the amount of rain (Figure A4g), due to the fact that it is a more critical element regarding the station layout. Correlations are generally lower when the distance between stations is greater. The higher the correlations are, the greater the reliability of the homogenisation and the filling of missing data will be. In particular, correlations should always be positive, at least at reasonable distances. Otherwise, as in our case, there are probably geographical discontinuities that produce climatic differences (for example, a mountain range can produce opposite precipitation regimes). This can be confirmed by the station map, where groups of similar variability would be located in different areas.

**Figure A5:**
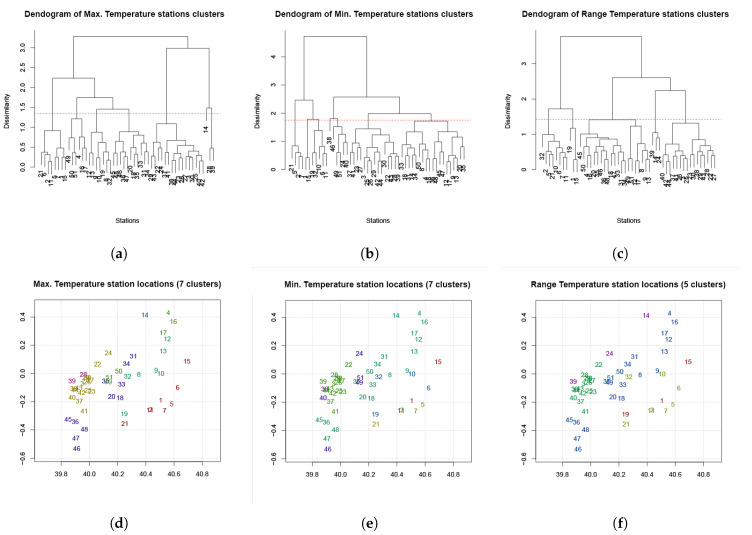
Dendrograms for maximum (left panel), minimum (central panel) and temperature range (right panel), showing the different clusters formed by the climatic stations of the three networks compared in this study (left), and maps showing the geographic location of the different clusters in the study area. (**a**) Maximum temperature cluster dendrogram. (**b**) Minimum temperature cluster dendrogram. (**c**) Range temperature cluster dendrogram. (**d**) Maximum temperature station locations. (**e**) Minimum temperature station locations. (**f**) Range temperature station locations.

**Figure A6:**
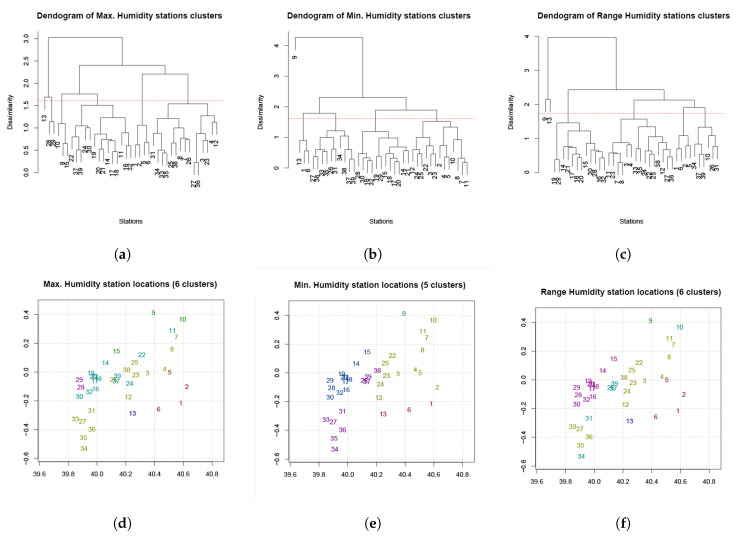
Dendrograms for maximum (left panel), minimum (central panel) and humidity range (right panel), showing the different clusters formed by the climatic stations of the three networks compared in this study (left), and maps showing the geographic location of the different clusters in the study area. (**a**) Maximum humidity cluster dendrogram. (**b**) Minimum humidity cluster dendrogram. (**c**) Range humidity cluster dendrogram. (**d**) Maximum humidity station locations. (**e**) Minimum humidity station locations. (**f**) Range humidity station locations.

**Figure A7:**
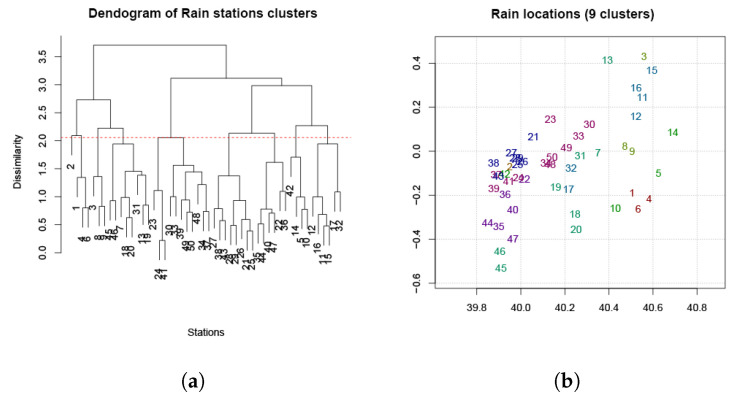
Dendrograms for rainfall, showing the different clusters formed by the climatic stations of the three networks compared in this study (left), and maps showing the geographic location of the different clusters in the study area (right). (**a**) Precipitation cluster dendrogram. (**b**) Precipitation stations locations.

In order to classify the climatic stations, we used cluster analysis to sort them into groups of similar variability. The resulting groups were plotted as dendrograms and maps of the stations with different colours, to locate groups of stations with similar variability (Figure A5 for temperature, Figure A6 for humidity and Figure A7 for precipitation). For temperature, the stations of the *SEnviro* network were scattered in other clusters, showing that the values observed in them are similar to those of the other networks. This situation occurred with the other phenomena studied, humidity and precipitation.

### 3.2. Standard Normal Homogeneity Test

After the exploratory study of the data, in this section, we analyse the results of the Standard Normal Homogeneity Test (SNHT) [33]. SNHT is appreciated for its power, simplicity and robustness. It is a single change-point detection test broadly embraced in climate time-series analysis for homogeneity assessment and change-point detection. Due to the large number of stations included in the analysis, we do not include all of them here, and we describe here the results from a weather station selected from each network, considering the completeness of the time series as a selection criterion.

**Figure A8:**
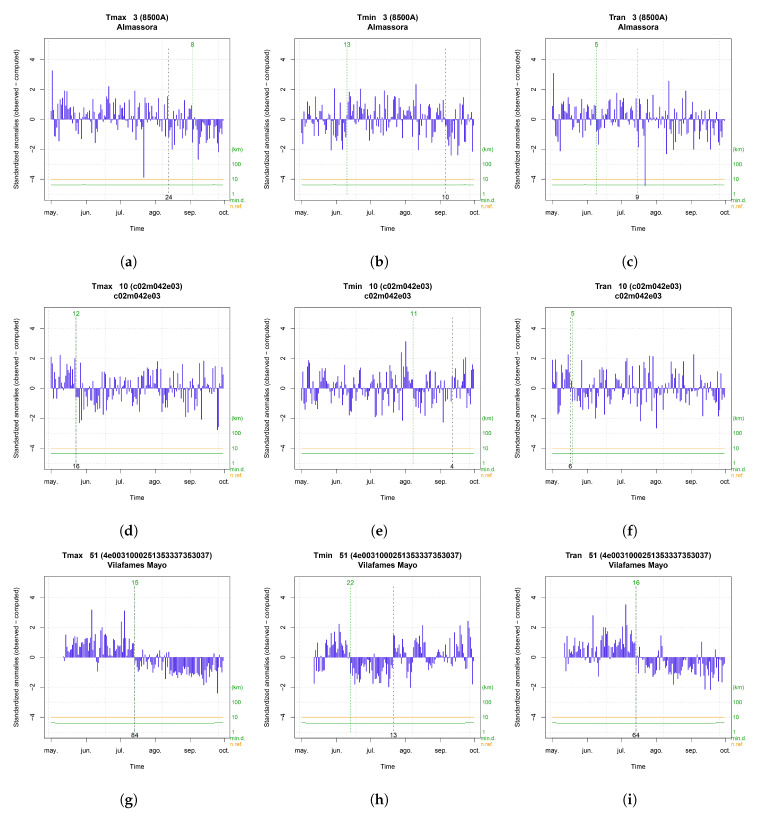
Standard normal homogeneity test for temperature. (**a**) Maximum temperature Almassora-*AEMET*. (**b**) Minimum temperature Almassora-*AEMET*. (**c**) Range temperature Almassora-*AEMET*. (**d**) Maximum temperature c02m042e03-*AVAMET*. (**e**) Minimum temperature c02m042e03-*AVAMET*. (**f**) Range temperature c02m042e03-*AVAMET*. (**g**) Maximum temperature *SEnviro2*. (**h**) Minimum temperature *SEnviro2*. (**i**) Range temperature *SEnviro2*.

**Figure A9:**
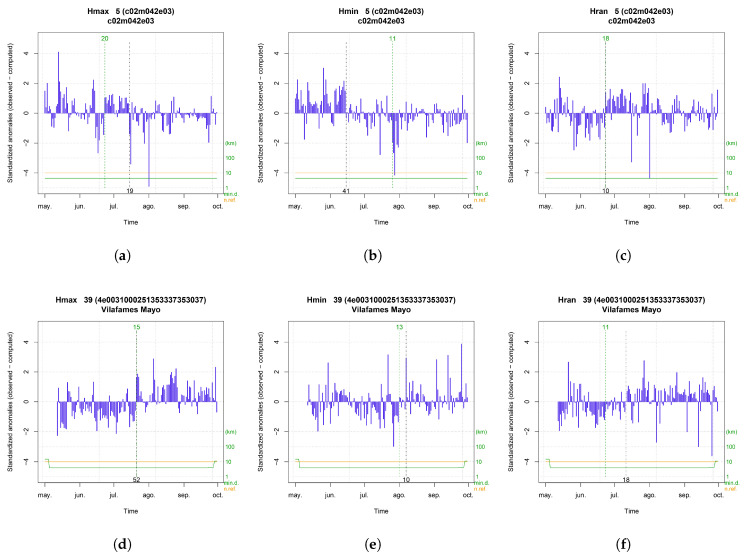
Standard normal homogeneity test for humidity. (**a**) Maximum humidity c02m042e03- *AVAMET*. (**b**) Minimum humidity c02m042e03-*AVAMET*. (**c**) Range humidity c02m042e03-*AVAMET*. (**d**) Maximum humidity *SEnviro2*. (**e**) Minimum humidity *SEnviro2*. (**f**) Range humidity *SEnviro2*.

**Figure A10:**
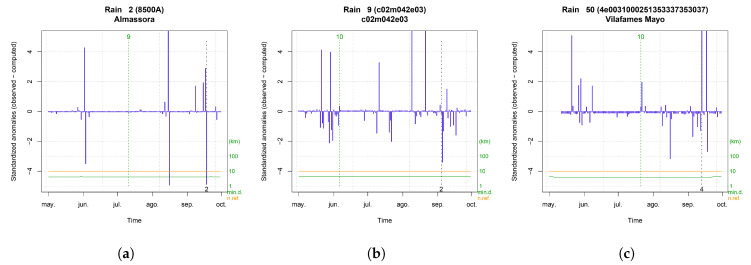
Standard normal homogeneity test for precipitation. (**a**) Precipitation Almassora-*AEMET*. (**b**) Precipitation c02m042e03-*AVAMET*. (**c**) Precipitation *SEnviro2*.

The graphs (Figure A8 for temperature, Figure A9, humidity and Figure A10 for precipitation) highlights the anomalies present in the original series, indicating the SNHT value. In the graphs, a green line is added where the SNHT is reached in staggered windows and a black line for the maximum SNHT in the entire series. Two additional lines at the bottom show the minimum distance of neighbouring data (in green) and the number of reference data used (in orange), both using the logarithmic scale of the right axis.

In all cases the variability is represented according to the measures, showing that the stations under study do not have much variation concerning the SNHT for the other stations (Figure A8, Figure A9 and Figure A10). Furthermore, as observed in the case of rain, the latter has less variation. In this case, since there are many values of 0 and points with large values, the graphs change, but this is because the data type is different from the previous ones.

If we focus on the case of temperatures (Figure A8), we see a stronger variability in the case of the *AEMET* and *AVAMET* stations throughout the entire period. Conversely, in the case of the *SEnviro* stations, we have long periods in the same measurement direction; that is, they behave more homogeneously concerning SHNT. Furthermore, the latter remain in the range of −2 to +2, thus improving homogeneity. In the case of *AEMET*, the values have more considerable variability, from −4 to +4, which shows less uniformity. For humidity, there is not as much variability between *SEnviro*’s nodes compared to those of *AVAMET* (Figure A9).

Finally, in this section, it is necessary to mention the SNHT case regarding precipitation (Figure A10). The range of values that we have in this case is similar among all types of nodes, *AEMET*, *AVAMET* and *SEnviro*. This gives us a better idea of the usefulness of these new nodes that are presented.

### 3.3. Adjusted Series and Applied Corrections

**Figure A11:**
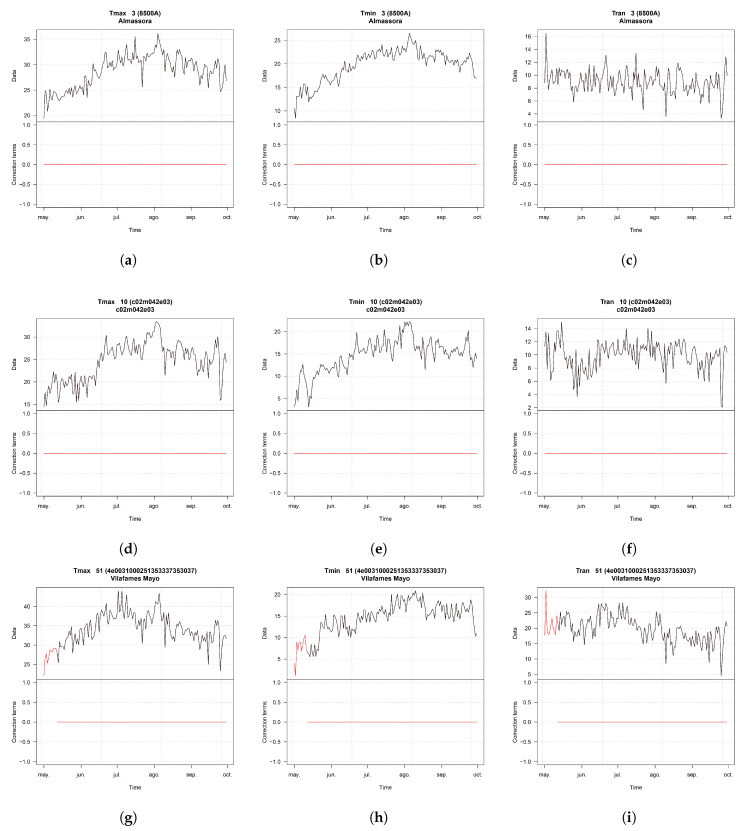
Reconstruction of missing temperature (minimum, maximum and range) observations. (**a**) Maximum temperature Almassora-*AEMET*. (**b**) Minimum temperature Almassora-*AEMET*. (**c**) Range temperature Almassora-*AEMET*. (**d**) Maximum temperature c02m042e03-*AVAMET*. (**e**) Minimum Temperature c02m042e03-*AVAMET*. (**f**) Range Temperature c02m042e03-*AVAMET*. (**g**) Maximum Temperature *SEnviro2*. (**h**) Minimum Temperature *SEnviro2*. (**i**) Range Temperature *SEnviro2*.

After the graphs of anomalies, we analysed the plots of adjusted series and applied corrections. Another of the advantages that Climatol offers is that it allows us to check if there are missing data in data series, as can be seen in Figure A11 for temperature maximum, minimum and the range. In this case, we can see that it works correctly as an initial idea to complete the series. Moreover, it can be used in any range of values.

**Figure A12:**
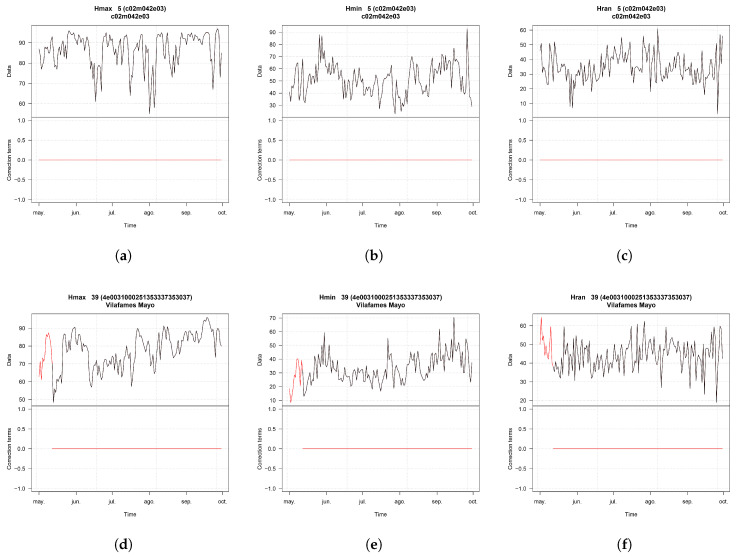
Reconstruction of missing humidity (minimum, maximum and range) observations. (**a**) Maximum Humidity c02m042e03-*AVAMET*. (**b**) Minimum Humidity c02m042e03-*AVAMET*. (**c**) Range Humidity c02m042e03-*AVAMET*. (**d**) Maximum Humidity *SEnviro2*. (**e**) Minimum Humidity *SEnviro2*. (**f**) Range Humidity *SEnviro2*.

**Figure A13:**
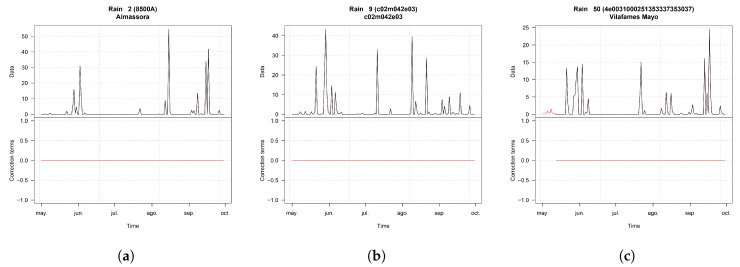
Reconstruction of missing precipitation observations. (**a**) Precipitation Almassora-*AEMET*. (**b**) Precipitation c02m042e03-*AVAMET*. (**c**) Precipitation *SEnviro2*.

Figure A12 and Figure A13 run in the same line but with regard to humidity and precipitation. There is a variation of ranges, but the behaviour of the data is similar. In all cases, there must not be too many missing data to perform a good evaluation.

### 3.4. Normalised Anomalies

**Figure A14:**
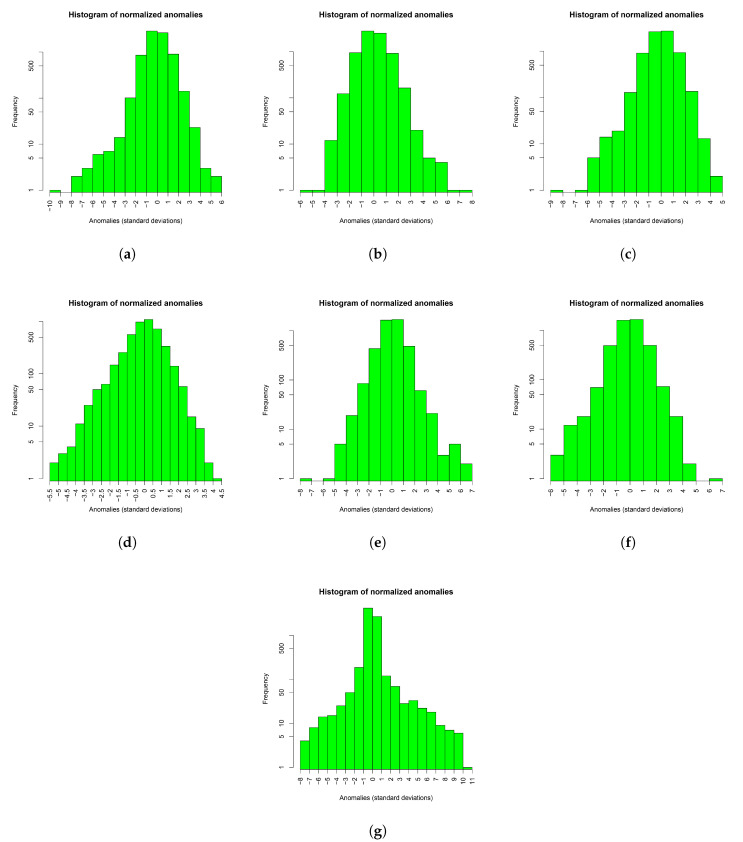
Temperature (minimum, maximum and range), humidity (minimum, maximum and range) and precipitation histogram of normalised anomalies. (**a**) Maximum temperature histogram of normalised anomalies. (**b**) Minimum temperature histogram of normalised anomalies. (**c**) Range temperature histogram of normalised anomalies. (**d**) Maximum humidity histogram of normalised anomalies. (**e**) Minimum humidity histogram of normalised anomalies. (**f**) Range humidity histogram of normalised anomalies. (**g**) Precipitation histogram of normalised anomalies.

In Figure A14, the anomaly histograms are presented. These help to choose appropriate thresholds to reject very anomalous data, assuming that they are errors and can be eliminated. This Figure A14 shows the distribution of anomalies for temperature, humidity and precipitation for the different seasons. Clearly, in all cases, they are distributed in a Gaussian way centred on the value 0. In short, it can be seen that the majority have few anomalies, many with values of 0. Some of the graphs have a minimal variation with negative values, similar to the data distribution histograms.

### 3.5. Station Quality/Singularity

This section shows a plot of station numbers according to their final Root Mean Squared Error (RMSE) and SNHT values. RMSE is calculated by comparing the estimated and the observed data in each series. A high value may indicate a bad quality of the series, but it could be caused by the station being located in a particular site with a distinct micro-climate. In any case, the homogeneous series from stations sharing the common climate of the region will tend to be clustered in the bottom left part of the plot.

**Figure A15:**
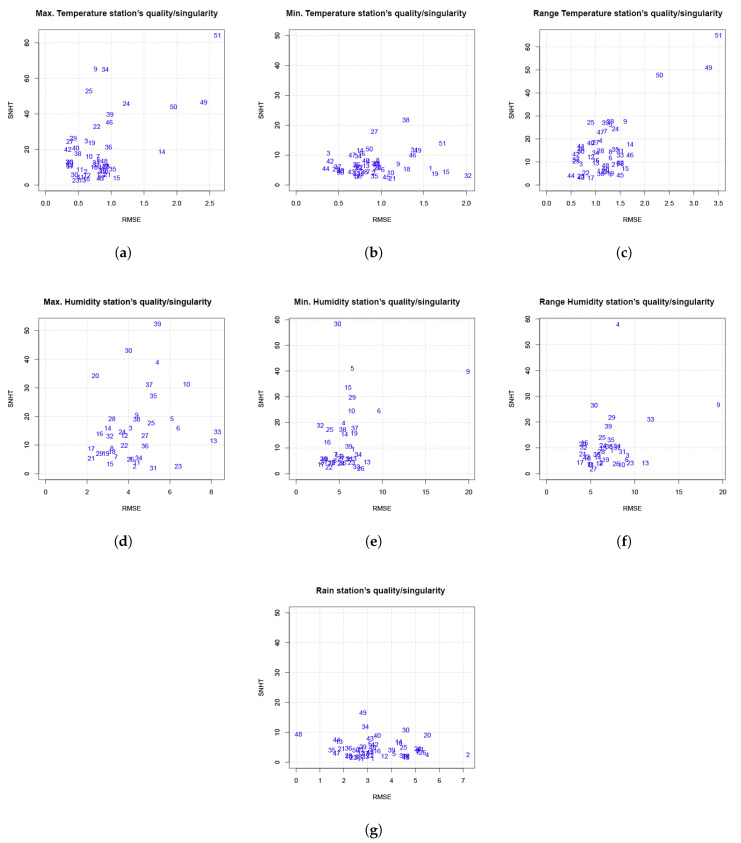
Quality/singularity of the temperature (minimum, maximum and range), humidity (minimum, maximum and range) and precipitation station. (**a**) Quality/singularity of the maximum temperature station. (**b**) Quality/singularity of the minimum temperature station. (**c**) Quality/singularity of the range temperature station. (**d**) Quality/singularity of the maximum humidity station. (**e**) Quality/singularity of the minimum humidity station. (**f**) Quality/singularity of the range humidity station. (**g**) Quality/singularity of the precipitation station.

On analysing Figure A15, and regarding the minimum temperature (Figure A15b), much variability was observed, but note that stations SEnviro1 and SEnviro3 are within the temperature range of the stations in the other two networks and that the RMSE is very low. In the case of the maximum temperature (Figure A15a), the three stations SEnviro1, SEnviro3 and SEnviro2 are the ones with more RMSE and those with almost the highest SNHT, so they have the most significant error. In addition, these stations 49-51 (*SEnviro*) have a lot of variability in range, as observed in Figure A15c. That is, the maximum and minimum temperature differences vary significantly and also have the highest associated error.

Concerning humidity, except c06m082e01 and c03m089e02 (the ones that show an apparent change of standardisation in the SNHT graphs, and in the charts (Figure A15d–f), they are not the ones that are missing), the rest are very grouped and so SEnviro1, SEnviro3 and SEnviro2 are not significantly different. In the maximum humidity (Figure A15d), as with the minimum (Figure A15e), there is a lot of variability in the data, and the RMSE is much higher. This is normal since the range of data is greater. In addition, SEnviro2 is the highest SNHT, whereas SEnviro1 and SEnviro3 are more moderate. Finally, if we study the humidity range (Figure A15f), the stations with the most significant error and variation are c02m042e02 and c03m089e02, which are seen to change from positive to negative in the SNHT graphs. In this case, it is important that the last three stations are within the range of the others.

A very important case, as throughout the study as a whole, is that of the amount of rain (Figure A15g). An interesting point is the fact that the SNHT is very low in all situations, SEnviro1, SEnviro3 and SEnviro2 are within the range, and the lowest RMSE is SEnviro1.

### 3.6. Similarity Analysis

Table 2 shows the values of the cross-correlation at lag 0 between the five closest *AVAMET* monitoring sites and the three monitoring locations of *SEnviro* for the different meteorological phenomena. The majority (75%) of the cross-correlations at lag h=0 are between 0.275 and 0.625, showing that the correlation between the chosen *AEMET* and the *SEnviro* climatic stations is moderate to low for the five phenomena compared. This may be a consequence of the spatial separation between the stations of the two networks and also the presence of micro-climatic conditions and local variability.

For maximum relative humidity, we found moderate cross-correlations at lag h=0, except for the pairs c04m122e01 (*AVAMET*)–SEnviro1 (*SEnviro*) and c05m120e01 (*AVAMET*)–SEnviro3, which show zero cross-correlation at h=0. The absence of cross-correlation is explained in part by the high number of missing values in the c04m122e01 (*AVAMET*) and c05m120e01 (*AVAMET*) stations and the different time variation of maximum relative humidity in the locations involved. Low cross-correlations and even negative cross-correlations at lag 0 are also observed for total precipitation. In this case, the explanation is the local behaviour of rainfall cells, which causes high spatial variation in this climatic phenomenon.

The cross-correlations at h=0 for total precipitation are shown in Table 3. For this climatic phenomenon, the correlations show high variability, ranging from values of −0.26 to 0.97. The highest correlation is observed in the pair c05m128e02 (*AVAMET*)–SEnviro1 (*SEnviro*), two stations that are relatively close in spatial terms. The negative correlation observed between the c05m105e08 (*AVAMET*) station and SEnviro2 (*SEnviro*) is a consequence of the presence of a highly influential observation, with high total precipitation values, but with the lower observation at c05m105e08 (*AVAMET*) and the higher value at SEnviro2 (*SEnviro*). This is one of the disadvantages of moment-related estimators [37].

For the maximum and minimum temperature (Table 4), we observe moderate to high cross-correlations at h=0 for most of the pairs of stations. In the case of maximum temperature, the correlations range between 0.49 and 0.82. The maximum temperature is achieved after noon, when wide areas have been warmed by sunlight and wind currents have homogenised such large areas, making maximum temperatures during early summer relatively stable in the study area, and with variability associated to topography and clouds. On the other hand, cross-correlations for minimum temperatures show a range between −0.23 and 0.62. The effect of topography and wind currents after sunset and during the night hours, as well as the presence of scattered rain showers, produces spatial variability in the minimum temperatures, and this is reflected in the variability of the cross-correlations. Again, outliers and influential observations were the cause of the negative correlations observed for the c05m105e08 (*AVAMET*) station with the SEnviro1 and SEnviro3 stations.

The analysis of the similarity of the time trends for the different climatological phenomena for the five *AVAMET* stations selected and the *SEnviro* stations shows that there is moderate to high similarity in the measurements obtained with the sensors used in the *SEnviro* stations. Higher similarities were not observed due to the presence of missing values and the presence of spatial and temporal variability inherent to climatic phenomena. The *SEnviro* system nevertheless has shown high potential for use in a more dense and wider low-cost network, capable of capturing the changes in weather phenomena that are of importance to farmers and the wine industry and for assessing the risk of other climate-related events such as floods and forest fires.

## 4. Conclusions

In this work, a reliability analysis of data generated by a low-cost meteorological station called *SEnviro* has been presented. Different units of these low-cost stations have been used to monitor fungal diseases affecting vineyards based on weather conditions. Three different phenomena were selected due to their strong relationship with the appearance of this type of diseases. The use of data obtained from *SEnviro* in agricultural areas along with the analysis of the data obtained by Climatol made it possible to perform a very complete analysis. The temporal behaviour of data obtained in the *SEnviro* stations was compared with those of *AEMET* and *AVAMET* in the same area in order to study their homogeneity and to identify possible difficulties and differences for possible implementation of a broader climatological network using the cheaper *SEnviro*-type sensors. In general, the data generated by low-cost stations do not produce a greater number of inhomogeneities compared to official and professional stations. Therefore, we can state that the results obtained from the Climatol study do not independently classify the data generated by the *SEnviro* stations.

We have also compared the similarity of the data obtained with the new *SEnviro* nodes and nearby professional stations (*AVAMET*). We found the existence of a significant cross-correlation of the data coming from the different networks, and that differences on a day-by-day basis are more likely to be a consequence of spatial variation, which is common in climatological phenomena. Significant similarity is seen between measurements obtained from the two networks, and we may conclude that the advantage of the *SEnviro* data, besides being reliable, is that they have a lower cost.

It should be noted that the province of Castelló is an area of great climatic variety due to its mountainous orography [29,30], with high temperatures in summer and very low ones in winter, and an important difference between the coast and the interior. In addition, the differences between the maximum and minimum temperatures are very large and far more so in the central area of the province. Thus, we may conclude that our results can be valid for other areas of Spain and even the European Community countries. All this is certainly valid for an agricultural area in the central zone of the province of Castelló, where the *SEnviro* stations were implemented. Nevertheless, we are sure that the low-cost *SEnviro* stations can be used for networks covering broader areas, resulting in a valuable source of climatic information for farmers at a low cost.

A longer period of monitoring to cover a wider range of weather conditions and to test the long-term stability of the sensors is suggested as future work. A follow-up study is proposed that will evaluate sensor performance using time series of at least one year and adding more low-cost sensors in a wide variety of different environmental conditions. Finally, emerging machine learning techniques will be explored to validate this type of low-cost sensor [38].

## Figures and Tables

**Table 1:** Information about the smallholdings where the *SEnviro* nodes are located.

Sensor Num.	Location (lat, long)	Area (metres)	Vine Variety
270…363036 (SEnviro1)	40.133098, −0.061000	20,000	Monastrell
4e0…353037 (SEnviro2)	40.206870, 0.015536	18,000	Merlot, cabernet, chardonnay and syrah
380…363036 (SEnviro3)	40.141384, −0.026397	15,000	Bonicaire

**Table 2:** Cross-correlations of maximum and minimum relative humidity between *SEnviro* stations and the nearest *AEMET* stations.

Phenomena		*SEnviro*	SEnviro1	SEnviro2	SEnviro3
	*AVAMET*				
	c04m122e01		0.27	0.27	0.00
	c05m128e02		0.54	0.61	0.72
Maximum	c05m105e09		0.47	0.53	0.45
	c05m105e08		0.30	0.30	0.47
	c05m120e01		0.27	0.27	0.00
	c04m122e01		0.73	0.92	1.00
	c05m128e02		0.53	0.48	0.39
Minimum	c05m105e09		0.62	0.72	0.64
	c05m105e08		0.37	0.42	0.56
	c05m120e01		1.00	0.92	1.00

**Table 3:** Cross-correlations of total precipitation between *SEnviro* stations with the nearest *AVAMET* stations.

	*SEnviro*	SEnviro1	SEnviro2	SEnviro3
*AVAMET*				
c04m122e01		0.46	0.200	0.10
c05m128e02		0.97	0.40	−0.32
c05m105e09		0.18	0.34	0.09
c05m105e08		0.76	−0.26	0.58
c05m120e01		0.46	0.02	0.26

**Table 4:** Cross-correlations of maximum and minimum temperature between *SEnviro* stations with the nearest *AEMET* stations.

Phenomena		*SEnviro*	SEnviro1	SEnviro2	SEnviro3
	*AVAMET*				
	c04m122e01		0.69	0.82	0.60
	c05m128e02		0.49	0.72	0.59
Maximum	c05m105e09		0.52	0.70	0.63
	c05m105e08		0.48	0.64	0.62
	c05m120e01		0.59	0.82	0.56
	c04m122e01		0.33	0.25	0.48
	c05m128e02		0.41	0.28	0.53
Minimum	c05m105e09		0.62	0.47	0.63
	c05m105e08		−0.22	−0.23	0.12
	c05m120e01		0.33	0.25	0.47
